# Automatic Target Detection from Satellite Imagery Using Machine Learning

**DOI:** 10.3390/s22031147

**Published:** 2022-02-02

**Authors:** Arsalan Tahir, Hafiz Suliman Munawar, Junaid Akram, Muhammad Adil, Shehryar Ali, Abbas Z. Kouzani, M. A. Pervez Mahmud

**Affiliations:** 1 Research Center for Modeling and Simulation, National University of Sciences and Technology, Islamabad 64000, Pakistan; atahir.mscse17@rcms.nust.edu.pk (A.T.); madil.mscse17@rcms.nust.edu.pk (M.A.); 2 School of Built Environment, University of New South Wales, Kensington, Sydney, NSW 2052, Australia; 3 Department of Computer Science, Superior University, Lahore 54700, Pakistan; junaidakram@superior.edu.pk or; 4 School of Computer Science, The University of Sydney, Camperdown, Sydney, NSW 2006, Australia; 5 School of Engineering, Deakin University, Geelong, VIC 3216, Australia; shehryarali317@gmail.com (S.A.); abbas.kouzani@deakin.edu.au (A.Z.K.); m.a.mahmud@deakin.edu.au (M.A.P.M.)

**Keywords:** deep learning, satellite images, YOLO, faster RCNN, SSD, SIMRDWN

## Abstract

Object detection is a vital step in satellite imagery-based computer vision applications such as precision agriculture, urban planning and defense applications. In satellite imagery, object detection is a very complicated task due to various reasons including low pixel resolution of objects and detection of small objects in the large scale (a single satellite image taken by Digital Globe comprises over 240 million pixels) satellite images. Object detection in satellite images has many challenges such as class variations, multiple objects pose, high variance in object size, illumination and a dense background. This study aims to compare the performance of existing deep learning algorithms for object detection in satellite imagery. We created the dataset of satellite imagery to perform object detection using convolutional neural network-based frameworks such as faster RCNN (faster region-based convolutional neural network), YOLO (you only look once), SSD (single-shot detector) and SIMRDWN (satellite imagery multiscale rapid detection with windowed networks). In addition to that, we also performed an analysis of these approaches in terms of accuracy and speed using the developed dataset of satellite imagery. The results showed that SIMRDWN has an accuracy of 97% on high-resolution images, while Faster RCNN has an accuracy of 95.31% on the standard resolution (1000 × 600). YOLOv3 has an accuracy of 94.20% on standard resolution (416 × 416) while on the other hand SSD has an accuracy of 84.61% on standard resolution (300 × 300). When it comes to speed and efficiency, YOLO is the obvious leader. In real-time surveillance, SIMRDWN fails. When YOLO takes 170 to 190 milliseconds to perform a task, SIMRDWN takes 5 to 103 milliseconds.

## 1. Introduction

Artificial intelligence (AI) is the field of computer science that aims to make machines intelligent. Such machines ideally respond like humans and in perceiving, understanding, and making decisions to solve problems [1,2,3]. AI covers a wide range of areas that mainly corresponds to human senses such as computer vision (CV), natural language processing (NLP) and controls and robotics. Computer vision is an area of computer science that tends to mimic human vision capabilities by understanding digital images and videos [4,5,6,7]. It uses various algorithms and optimization techniques to analyze images. CV is an interdisciplinary field which spans machine learning, pattern recognition, robotics, mathematics and probability. Machine learning (ML) is the subfield of AI that learns from data rather than programming explicitly [8]. ML is highly used in tabular data. To understand images and videos, a deeper and complex model is required. Researchers have found that neural networks (NN) are exceptionally good at consuming large amounts of data (e.g., images and videos) and making sense of it. With the use of neural networks in CV, researchers were able to solve complex problems such as image classification, object detection, object recognition and instance segmentation optical character recognition. Computer vision is also involved in the detection and analysis of objects in images using deep learning algorithms. By solving the mentioned problems, CV has made an impact in fields such as medical imagery analysis, satellite imagery analysis, autonomous driving, human activity analysis, document analysis, etc. [9,10,11,12,13].

Object detection has been one of the primary tasks in computer vision and active research for several years. The main goal of object detection is to find an instance from images and videos [14,15,16]. In the context of CV, object detection is the technique of detecting objects of interest (e.g., human, cat, dog, cycle, etc.) at specific location in image (via a bounding box) [17,18,19]. Object detection has many applications in the field of artificial intelligence and computer vision including robot vision, security, and surveillance and augmented reality. Object detection has two categories. The first type of detection is to find generic categories (human, cat, etc.) and second type target specific instances such as the president’s face. First, researchers have focused on the detection of specific categories but for the past several years people are working on the detection of generic object detection. In object detection, objects refer to a thing that can be touched and seen.

Object detection basically also incorporates another CV algorithm, i.e., image classification (in which the objective is to only predict whether an image contains an object of interest or not). In other words, object detection algorithms first try to find whether an object of interest is present in the image. If yes, the next step is to find the coordinates of the object and draw a bounding box around it. Meanwhile, drawing a bounding box around an object is a regression problem itself. Drawing the bounding box is actually guessing the pixels which enclose the object of interest.

**Figure 1:**
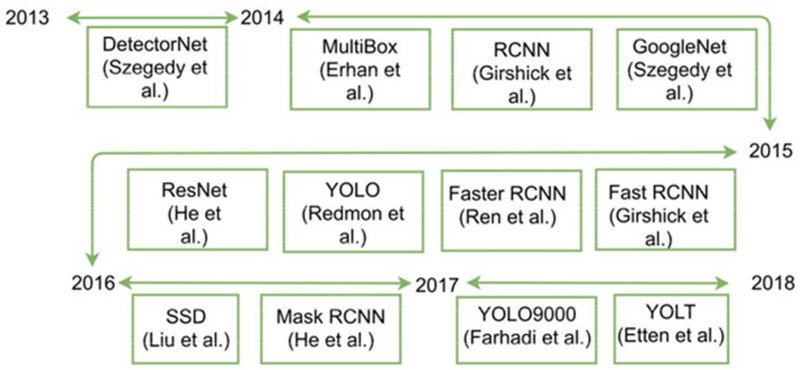
Major contributions of object detection frameworks and convolutional neural networks.

Figure 1 shows the generic object detection frameworks, which use deep learning for detection. Object detection has many challenges which are discussed in the next section.

In this research paper, we are going to address the object detection problem in satellite imagery. Object detection in satellite imagery has its own challenges and its own plan of action to solve those challenges.

### 1.1. Satellite Imagery

Object detection is influenced by two sorts of challenges: the first is an accuracy related problem, and the second is efficiency related. Former one is the wide range of intraclass changes, and the later one is the vast number of object categories. Accuracy of object detection models rely largely on these two factors. We begin with intraclass variance, which is split into two categories—imaging condition and intrinsic factor. For each category, such as chairs, objects appear with a lot of variations, e.g., positions, color, texture and a material’s reflection property. The appearance of objects is also affected by the surroundings and lighting conditions. Deformation, lighting, position, occlusion, scale, blur, background clutter and shading are all factors that affect the appearance of objects. As a result, most researchers concentrate on structured object categories [20,21,22,23].

Another concern is the computational power when it comes to object detection. For the optimal detector, devices have less processing power and storage space. When there are a high number of objects in a single picture with too many categories, efficiency suffers. Scalability of models is also an issue in real-time application, since the model must deal with unexpected and unfamiliar situations. Object detection models require huge, labeled data. Labeling object detection dataset is one of the most time consuming and also very expensive tasks if performed by experts of relevant domains.

### 1.2. Satellite Imagery Acquisition for CV Applications

**Figure 2:**
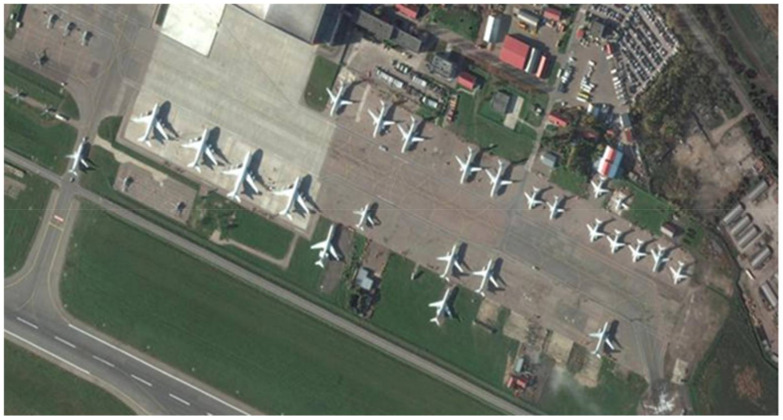
This satellite image is taken by Google Earth. It is an open-source platform for collection of satellite images. This image contains objects of interest, i.e., airplanes.

Satellite images are acquired from commercial or government satellites. Researchers heavily rely on open-source satellite imagery databases that are updated frequently. We can obtain satellite imagery in range of resolution and historical imagery from open-source satellite imagery providers such as Google Earth Pro and Bing Maps. More detailed discussion on resolution and sources of satellite imagery will be discussed in upcoming subsections. We can detect small objects such as aircraft, vehicles and ships from the highest resolution images (e.g., around 0.5 m/pixels). We can detect large size objects, e.g., airports, roads and large buildings from medium resolution (e.g., around 1 m/pixels). Figure 2 shows the image of a satellite taken by Google Earth.

### 1.3. Object Detection in Satellite Images

Object detection in satellite images is a task to find a specific instance of one or more categories, such as an airplane, and locate the position of objects. In satellite image analysis, object detection has always played a vital role in many applications, such as geographical information systems, environmental monitoring, precision agriculture and urban planning. For this purpose, we need robust algorithms for automatic object detection on satellite images. Deep learning provides an accurate and efficient solution for the purpose of detecting objects in the satellite images.

### 1.4. Challenges in Satellite Images

The classifiers show poor results on satellite images because of different conditions. In spatial resolution, objects are of very small size and are densely clustered in satellite images rather than the prominent and large object and for small objects such as cars, an object is only ~15 pixels in high-resolution images. In rotation invariance objects in satellite imagery have many orientations (for example ships have any orientation ranging from 0 to 360 degrees). In training example frequency, there is a relative dearth of data in satellite imagery and objects are not clearly visible in shape. In ultra-high-resolution, images are of very high resolution (hundreds of megapixels) but most algorithms take in- put images with a few hundreds of pixels. Up sampling the image means the object of interest becomes large, dispersed and not feasible for standard architecture and down sampling the image can change the object shape. Temporal (time of day/season/year) seasonal differences and time of day also affect satellite images. Therefore, it is difficult for a classifier to detect objects from conventional datasets due to mentioned reasons on satellite images. For this, we need a specialized kind of data for satellite images for the processing that is computationally less expensive and time efficient.

### 1.5. Problem Statement

Object detection is a very important, fundamental and challenging problem in satellite images because objects are densely clustered, small and multi-oriented. Therefore, to detect and localize small objects in satellite images is a primary problem. For this purpose, we have made a custom dataset with low-resolution images containing objects (such as small size aircraft) in images to achieve good accuracy with the usage of low computational power because the dataset of low-resolution images helps to reduce the training time. We have performed analysis of different object detection pipelines using custom dataset in terms of speed and accuracy.

## 2. Contributions

The main application of our work is in the defense sector. Within specific areas of operations, we use sophisticated algorithms to detect, categorize and identify airplanes, surface vessels and space objects. On a daily basis, incursions into airspace and its approaches are carried out for illegal objectives against sovereign territories. Our method marks the beginning of the creation and maintenance of an up-to-date and comprehensive picture of air, space and surface operations utilizing satellite photos and, in the future, drones.

- We created a dataset with object aircraft of satellite images and apply preprocessing techniques on the dataset for testing and training.
- We increased the number of objects in a dataset for achieving accuracy and also decreased the computational cost using low-resolution images.
- We carried out a survey of the existing approaches/algorithms used for detection of objects (aircraft) in satellite imagery.
- A comparison of performance of major algorithms (in terms of execution speed and accuracy) for detection and classification of aircraft in satellite imagery using custom dataset was performed. There are five sections to this study.

The first portion is dedicated to the introduction. Section 2 deals with related work. Methods for object detection are explored in Section 3. The results are discussed in Section 4, and the conclusion is discussed in the last section.

## 3. Related Work

In recent years, different problems are solved relative to object recognition in satellite imagery using machine-learning and deep-learning techniques. Three pipelines, which provide real-time solutions, are Faster-RCNN, SSD and YOLO. YOLO (you only look once) is state-of-the-art real-time object detection framework based on a CNN (convolutional neural network) algorithm and takes an image of 416 × 416 resolution. Faster RCNN is a state of the art framework based upon the region proposal algorithm, and takes an image of 1000 × 600 resolution. SSD (single shot detector) framework extracts feature maps through different layers and applies CNN filters to detect an object and runs on either 300 × 300 or 512 × 512 pixels per image. Jamie et al. [24] applied dense labeling on ISPRS (International Society for Photogrammetry and Remote Sensing) Vaihingen and posts dam benchmark datasets [25], which contain high-resolution images followed by CNN for fine-tuning and hits state-of-the-art accuracy. Yang Long et al. [26] targeted remote sensing and localization by employing region-based techniques and classification algorithms, and showed better results for object localization. However, the latency was high due to the region-based method and did not cover the large area (40 s covered area of 1280 × 1280 pixels). The work of Volodymyr Mnih and Geoffrey E. Hinton [27] used segmentation and post-processing techniques and gave reliable results of automatic road detection in satellite imagery, but it was proven to be slower for segmentation. In satellite images, J. Khan et al. [28] suggested automatic object recognition using the edge boxes technique.

Using the YOLO framework, Junyan Lu et al. [29] presented a technique for vehicle detection. Lu Zhang et al. [27] demonstrated a deep learning-based oil tank detector for high-resolution satellite data. Their proposed method comprises three parts, which first select candidates and apply feature extraction and classification. Marcum et al. [30] presented a method SAM (surface-to-air missile sites) to search objects in high-resolution imagery using sliding window techniques and covered the large area of earth’s surface. They used CNN architecture with five convolutional layers along with three fully connected layers. At last, a SoftMax layer is applied, which is used to calculate the probability distribution among all object classes. If the object is of hundreds of meters of size, then this approach performs better results rather than smaller objects. Zhang et al. [31] used Deep Residual U-Net architecture with fewer parameters for road extraction and obtained better performance.

Radovic et al. [32] presented object recognition research work in aerial images using a CNN. They just used the YOLO framework to localize objects in satellite images and gave 95% accuracy.

Van et al. [33] proposed a new method, YOLT (you only look twice), which evaluates the native resolution of images, processed buildings, and vehicles at a rate of 30 km^2^ and airports at a rate of 6000 km^2^ per minute. They proposed a model YOLT (you only look twice) that performs detection on satellite imagery at a rate of 0.5 km^2^/s and detects objects with different scales and sizes. YOLO cannot detect objects less than 32 pixels, but YOLT detects only five pixels and is also localized with good confidence. Van [34,35,36,37] also proposed a new model named SIMRDWN, which is an updated version of YOLT along with faster RCNN and SSD. This model evaluates the satellite imagery at a rate of 0.2 km^2^/s for vehicle objects. Frameworks based on CNN, which can detect objects in real-time are higher in performance and less computationally expensive compared with other machine learning algorithms. Without the implementation and development of artificial intelligence within aerial applications, the automation is not executable in real-time due to complexities and computational cost. Thus, deep learning-based pipelines, which use CNN algorithms, provide solutions for the detection and classification of objects from data in real-time. From a few years, these algorithms have shown reliable results for image classification and object detection due to their speed and high accuracy. In this study, the CNN algorithm used advanced GPU technology for processing in real-time. For many decades neural network algorithms perform parallel processing due to parallel computing hardware. Therefore, neural network structure is parallel and has been made according to the architecture of GPU which consists of many hundred cores and performs multiple tasks simultaneously. Due to the advantage of this parallelism, software has low latency, throughput and computational cost to perform classification and detection. The computationally expensive and highly complex feature extraction algorithms related to traditional machine learning methods were used in the recent past to extract low-dimensional feature vectors for clustering and vector quantization. Their inability to be parallelized, resulted in higher computational cost and time for processing. We have made a custom dataset of satellite imagery containing object aircraft, because the public dataset has limitations in resolution, usability and is not suitable for our specific problem, and we then run the models on this dataset. The methodology and results of these models are discussed in the next sections.

## 4. Methodology

Dataset creation is one of the crucial steps of the whole object detection pipeline as performance and accuracy of the model are highly dependent on the dataset. It is the most important aspect in assessing and analyzing the effectiveness of various algorithms. The internet allows larger photos with a great number of categories to be used to capture the diversity and complexity of items. The rise of large-scale datasets including millions of images has played a key role in enabling remarkable object detection performance.

**Figure 3:**
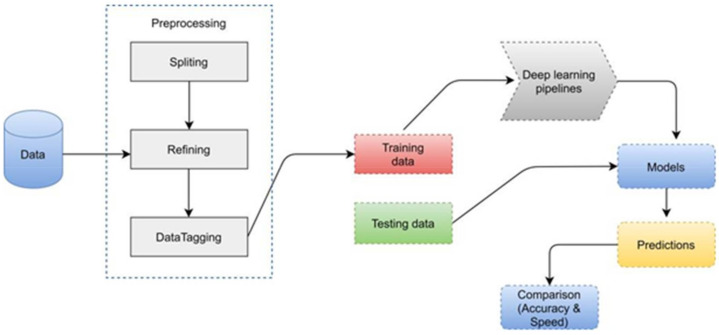
Pipeline of object detection implemented in this research. It starts from data collection followed by model training and making predictions.

We used Google Earth to acquire satellite photos with a resolution of 1920 × 1080 pixels. Real-time surveillance satellite images are very hard to find as they are usually classified. Thus, Google earth is the best option to find satellite images of aircrafts. Therefore, we tried to find as many images of aircrafts as we could. The dataset should be bigger but we are restricted to what we can find. After the collection, we divided the images into 550 × 350 resolution to reduce the training time. Manually we then removed all images, which contained no objects. We have 442 images with 2213 objects of aircraft in our dataset. After that, we used the labeling tool [38,39,40] for tagging images. We used the Python language to transform the data into standard architecture after tagging/labeling it. After that, we performed training on our custom dataset and checked results discussed in the next section. Figure 3 represents the methodology diagram of our complete work.

### 4.1. Network Architectures

#### 4.1.1. Faster RCNN

**Figure 4:**
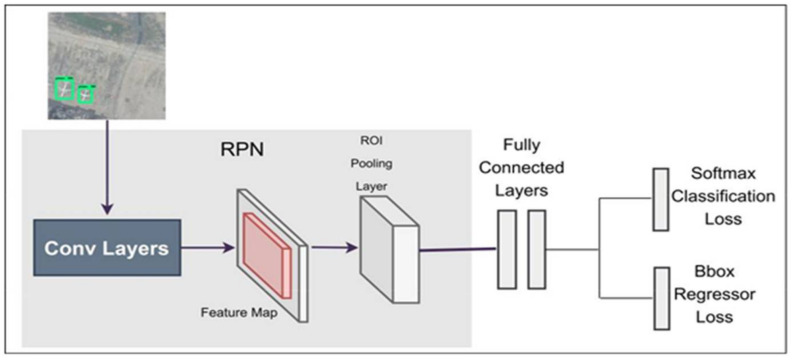
Overview of Faster RCNN network layers.

Faster RCNN [41,42,43] is a two-stage detection framework, which includes the generation of regions in one-step and the second step includes classification and localization of objects. Fast RCNN depends upon external region proposals and its detection process is fast. Recent work shows that CNN has the ability to localize objects in CONV (convolutional) layers and this is weak in fully connected layers. Therefore, CNN was replaced by the selective search for producing regional proposals. They proposed an accurate and efficient region proposal network (RPN) for producing regions proposals by replacing selective search. They divide the framework into two modules, first is RPN for the generation of region proposals and second is fast RCNN for classification and localization of objects. In faster RCNN, a large number of convolutional layers shared by RPN and last convolutional layers is responsible for classification and localization of objects via bounding boxes. Network architecture of faster RCNN is shown in Figure 4. RPN generates k n × n anchor boxes on features extracted by CONV layers with different aspect ratios and scales. Each n × n anchor is converted into a low dimensional vector such as 512 for VGG (visual geometry group) and 256 for ZF, which is fed into two fully connected layers consisting of bounding boxes regressor layers and object classification layers. The size of region proposals generated from faster RCNN is the same as the regions proposals from fast RCNN. RPN enables efficient region proposals computation and shares features with the fast RCNN, because RPN is a type of fully convolutional network. Faster RCNN is purely used CNN for feature extraction rather than handcrafted features (Figure 4). With VGG16 [44] model, faster RCNN gives 5 fps on GPU and achieves object detection accuracy on PASCAL VOC [45,46] dataset using three hundred proposals per image. With the rapid development of faster RCNN, Lenc et al. [47] studied the role of generation of region proposals through selective search and generation of region proposals through CNN and claimed that CNN based RPN contains less geometric information for object detection in the CONV Layers rather than FC layers.

#### 4.1.2. YOLO

**Figure 5:**
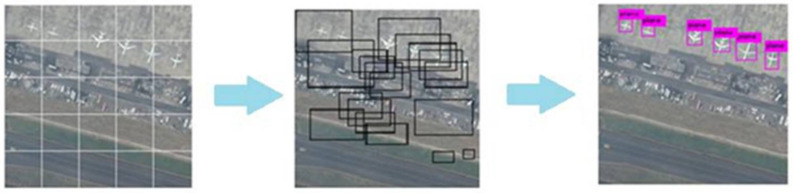
YOLO model divides image into S × S grid and calculates confidence scores with B bounding boxes and predictions are enclosed into (S × S) × (B *5 + C) tensor.

YOLO (you only look once) [48,49,50,51] is a real-time object detector based on convolutional neural networks introduced by Redmon et al. After some time, Joseph Redmon and Ali Farhadi published YOLO v2 [52], a new version with improved performance and speed. The most recent version is YOLO v3 [53], which was proposed by Joseph Redmon and Ali Farhadi to enhance speed and accuracy by increasing the number of layers in the design. Due to its architecture, YOLO offers several benefits over standard algorithms. The conventional technique included generating suggestions using area proposal networks and then applying CNN to these ideas. YOLO uses end-to-end training and increases real-time speed to achieve the high average precision.(Figure 5) The image is divided into S × S by YOLO, and if the item’s center falls within the grid, the object is predicted by this grid. Each grid cell predicts confidence scores with class probability and B bounding boxes. The confidence score tells the accuracy of the box on predicted objects. Confidence is pr(object) × IOU (pred, truth) and intersection over union (IOU) tells the evaluate matrix which is used to measure the accuracy of the object detector. If the object is not present, the confidence is equal to zero if present then it is equal to the probability of the object with intersection over the union between the ground truth and predicted box. Each bounding box consists of four values (x, y, w, h, confidence), where x and y are the center points of objects relative to the grid and w and h are the width and height correspondence to the whole image. The confidence tells the IOU between ground truth and predicted box. Each grid cell is also predicted by the conditional probability and at test time it is calculated with multiplication of bounding boxes confidence scores, which gives us object class confidence scores.

The GoogLeNet [54] model for image categorization influenced the YOLO network design. There are 24 convolutional layers and two fully linked layers in this network. Instead of inception modules, they just employ 1 × 1 reduction layers followed by 3 × 3 convolutional layers.

#### 4.1.3. SSD

**Figure 6:**
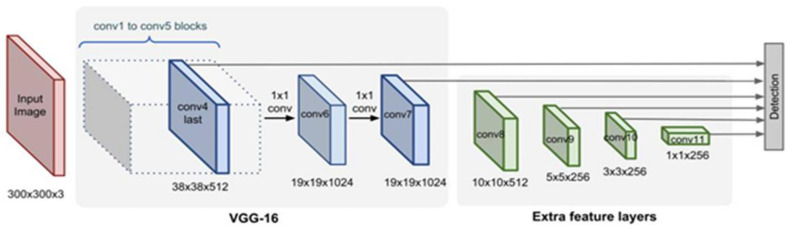
SSD uses feature layers at the end of base network, which predicts the class score with four offset values to default boxes.

SSD (single shot detector) [55] is based on feed-forward CNN and produces a fixed size of bounding boxes and scores through non-maximum suppression techniques for the presence of objects in those boxes. They use simple CNN for classification, named base network, and add convolutional features layers at the end of base network. They use VGG-16 for the base network. These layers help predictions of object detection at multiple scales and decrease in size progressively (Figure 6). Each feature layer produces different detections and different according to the convolutional model. With the usage of convolutional filters, each layer produces a fixed set of predictions. The predicting parameter of detection 3 × 3 × p (kernel) used for feature layer m × n produces score value and four coordinates of object location. At each location, output values are calculated to a default box location to each feature map position. For each box, SSD calculates a class with four offset values relative to the original box (Figure 6).

#### 4.1.4. SIMRDWN

YOLO, faster RCNN and SSD perform detection on standard resolution. Satellite images are of very high resolution and these architectures fail to perform detection on this resolution. Due to limitations of these architectures, Van et al. [56] proposed a new pipeline SIMRDWN (satellite imagery multiscale rapid detection with windowed networks), which evaluates high resolution of images, processed buildings and vehicles at a rate of 30 km^2^. The proposed model, which performs detection on satellite imagery at a rate of 0.2 km^2^/s and detects objects with different scales and sizes. YOLO cannot detect objects less than 32 pixels, but SIMRDWN detects only 20 pixels and is also localized with good confidence. They extend the YOLO, integrate the c libraries with python, and make a new model for satellite images. For data preprocessing it is very difficult for researchers to train and test models in C, but it is easy in python as a lot of support and libraries are available for machine learning. They reduce coarseness in features and accurately detect dense objects through their model with many changes in architecture. They implement network architecture with 22 convolutional layers with factor 16 and a down-sampling image of 416 × 416 makes 26 × 26 grid cells. SIMRDWN architecture is inspired by YOLO and updates many parameters for correct detection of small and dense objects. However, there is one drawback of dense grid 26 × 26, which is diffusion of large objects such as airports. They use a pass-through layer that concatenates the 52 × 52 layer with the last convolutional layer for this purpose and their architecture detects large objects as well as small objects. The leaky rectified linear unit (ReLu) [57,58,59] is used for the last of each convolutional layer for activation of output and for non-linearity in feature extraction. The final layer is responsible for output, which is the combination of B bounding boxes and C class probabilities and prediction tensor has size Nf = N boxes × (N classes +5), where N classes is the total number of object classes and Nboxes is total boxes per grid (Table 1).

### 4.2. Network Training

#### 4.2.1. Faster RCNN

For training of faster RCNN we placed all coordinates of objects in a single text file containing height, the width of image, center points of objects, height and width of objects and object class.

(1) $$\left(himg,\;wimg,\;x,y,w,\;h,objectclass\right)$$

When training faster RCNN use anchors and multiple proposals, K is generated at each sliding window location. Reg layer has 4K boxes with coordinates and the cls layer has a 2k score with a class probability of object or not object. The K parameter refers to k boxes, which are called k anchors. At each sliding window, they use k = 9 with three scales and three aspect ratios and at convolutional feature map W × H, almost WHK anchors are generated. Faster RCNN uses CNN for features computed on a single scale image to handle the multiscale problem based on anchors. This has benefits for feature sharing and less cost for addressing multiscale. For training, they assign binary labels for each object in terms of the presence of the object or not. They assign a positive label for anchors. There are two ways of calculating anchors. First, one takes those anchors whose intersection over union is high with ground truth box and second takes those anchors whose intersection over union is greater than 0.7 with ground truth box and ground truth boxes assign labels to multiple anchors. Thus, the second condition is not appropriate for good prediction of anchors. Therefore, they use the first condition, which has the highest IOU with ground truth box and assigns positive labels to anchors. They also assigned a negative label to anchors, which has the lowest IOU like 0.3 with ground truth box. Anchors are not involved in training, which has neither a negative nor a positive label. For this reason, faster RCNN uses loss function:

(2) $$L\left(\left\{p_{i}\},\{b_{i}\right\}\right)=\frac{1}{M_{\mathrm{cls}}}\sum_{i}L_{\mathrm{cls}}\left(p_{i},p_{i}^{*}\right)+\lambda \frac{1}{M_{\mathrm{reg}}}\sum_{i}p_{i}^{*}L_{\mathrm{reg}}\left(b_{i},b_{i}^{*}\right)$$

Here, $p_{i}$ is the probability of anchors at i  and represents the index of anchors in training. The $p_{i}$ represents 0 if ground truth is negative and it shows positive if ground truth is positive. bi is the vector of four coordinates (Bounding Boxes) at object location and attached with positive anchors. The $L_{cls}$ is the classification loss and it depends upon the classes of objects. The $L_{reg}$ is the regression loss function. If the object is present then this term $p_{i}$ $L_{reg}$ activates the regression offset and if not present then this is not activated. The output of regression layers is $f_{big}$ and classification layers is $f_{big}$ respectively. The $M_{cls}$ and Mreg are the parameters, which are used for balancing l. By default, the used l = 10 and their experiments show both reg and cls are roughly equally weighted and insensitive. Faster RCNN supports image-centric strategy, therefore we use this strategy for training the neural network. Every mini-batch becomes apparent from a single image, which contains many negative and positive anchors. When they use all anchors then a loss will be calculated but it dominates the negative samples due to biases. For this reason, they use 256 anchors and set the ratio of positive and negative samples 1:1 for the computation of the loss function of each mini-batch. If the image has fewer than 128 samples then they pad the mini-batch with a negative one. We used 2213 objects of the airplane for training with learning rate 0.0001 and used pre-trained weights.

#### 4.2.2. YOLO

We transformed the dataset into the standard YOLO architecture for YOLO training. The dataset is divided into two sections. The collection is divided into two parts: pictures in JPEG format and labels in text format. Evert text files are saved in accordance with pictures that contain object annotations in the format:

(3) $$\langle \;object\_class\;\rangle \langle x,y,w,h\rangle$$

where *x* and *y* are the object’s center coordinates, and w and h are the object’s width and height, respectively, with relation to the picture and name of the object class. For training, the YOLO input dimension is 416 × 416 × 3; however, one should keep in mind that the image size should not be too big or important information will be lost.

**Figure 7:**
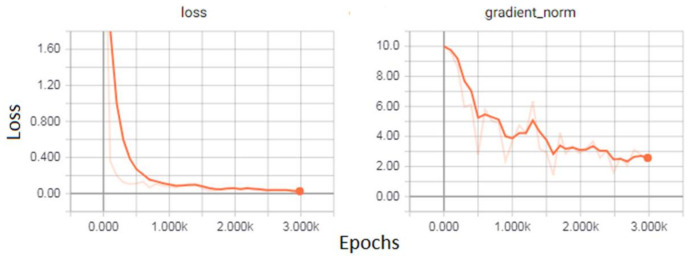
Loss after training of model faster RCNN.

YOLO optimizes the model using sum squared error, however it does not coincide with the aim of maximizing average precision (Figure 7). First, it assigns equal weights to classification and localization error, which is not appropriate for training. However, the model also produces instability, because many grid cells do not contain objects and confidence becomes zero and also overpower the gradient. For this, they decrease the loss of coordinates of boxes that have no objects and increase the loss of those coordinates having objects. They use lcoord and lnoobj parameters to handle this problem and set lnoobj = 0.5 and lcoord = 5.

Sum squared error assigns equally weights error to large and small boxes. YOLO predicts square root of width and height of bounding boxes rather than height and width directly because without square root small deviations in large boxes show less than small boxes.

At the training, YOLO uses one bounding box for each object and calculates IOU with the ground truth and uses the best bounding box predictor. Each predictor performs better and improves recall at different aspect ratios, sizes or class of objects. During training YOLO uses this loss function for optimization:

(4) $$\lambda_{\mathrm{coord}}\sum_{i=0}^{S^{2}}\sum_{j=0}^{B}1_{\mathrm{ij}}^{\mathrm{obj}}[{\left(x_{i}-{\hat{x}}_{i}\right)}^{2}+{\left(y_{i}-{\hat{y}}_{i}\right)}^{2}]\lambda_{\mathrm{coord}}\sum_{i=0}^{S^{2}}\sum_{j=0}^{B}1_{\mathrm{ij}}^{\mathrm{obj}}[{\left(\sqrt{w_{i}}-\sqrt{{\hat{w}}_{i}}\right)}^{2}+{\left(\sqrt{h_{i}}-\sqrt{{\hat{h}}_{i}}\right)}^{2}]\;+\sum_{i=0}^{S^{2}}\sum_{j=0}^{B}1_{\mathrm{ij}}^{\mathrm{obj}}[{\left(C_{i}-{\hat{C}}_{i}\right)}^{2}]+\lambda_{\mathrm{coord}}\sum_{i=0}^{S^{2}}\sum_{j=0}^{B}1_{\mathrm{ij}}^{\mathrm{noobj}}[{\left(C_{i}-{\hat{C}}_{i}\right)}^{2}]\;+\sum_{i=0}^{S^{2}}1_{\mathrm{ij}}^{\mathrm{obj}}\sum_{c\in \mathrm{classes}}[{\left(p_{i}\left(c\right)-{\hat{p}}_{i}\left(c\right)\right)}^{2}]$$

When an item is not present in a grid cell, the loss function examines classification error, and for bounding boxes, it assesses coordinate error when the predictor is in effect for the ground truth box. We use 1100 epochs to train the model, with batch = 64, decay = 0.0005, and momentum = 0.9.

#### 4.2.3. SSD

**Figure 8:**
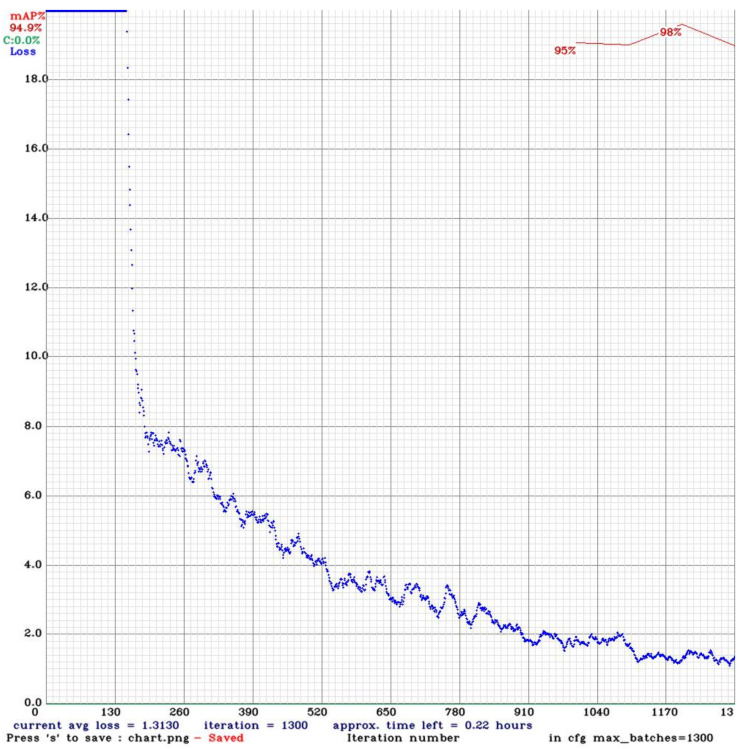
Loss function after training the YOLO Model.

SSD uses the training procedure same as multi box objective [60,61,62,63] and handles multiple object categories. It chooses different aspect ratios and scales for default boxes and combines the results after processing the images with different sizes. It uses a single network for prediction by utilizing features from different layers and shares parameters alongside all object scales (Figure 8). SSD uses lower and upper feature maps for detection because lower feature maps improve semantic segmentation quality and capture the fine information of input objects. The loss function is a combination of localization loss and confidence loss for SSD.

(5) $$L\left(x,c,b,g\right)=\frac{1}{K}\left(L_{conf\;}\left(x,c\right)+\alpha L_{loc\;}\left(x,b,g\right)\right)$$

where K represents the number of match default boxes. If K=0 the SSD considers loss is equal to zero. The localization loss is the same as an L1 loss between ground truth $\left(g\right)$ and predicted box $\left(b\right)$. During training, when possible, default boxes are large then these boxes become negative and cause a significant imbalance between negative and positive training examples. SSD solve this problem using the highest confidence loss and set ratio between negative and positive 1:3. This technique helps in stable training and faster optimization.

#### 4.2.4. SIMRDWN

**Figure 9:**
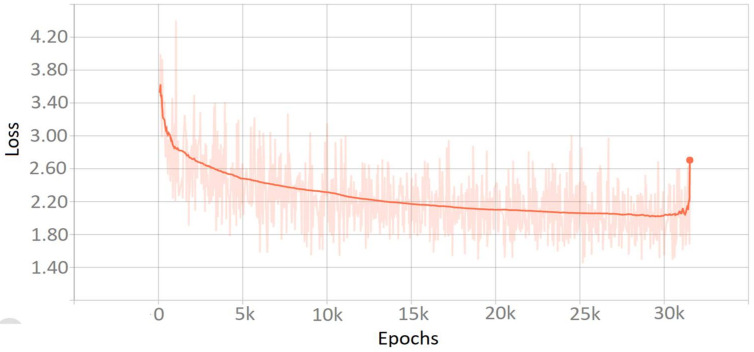
Loss function after training the SSD Model.

We assemble a dataset of satellite images from a variety of sources, manually annotating and placing anchor boxes on the things of interest. The collection is separated into two sections: photographs in JPEG format and text labels (Figure 9). Evert text files are saved in accordance with images including object annotations, and the annotation format is as follows:

(6) $$\langle \;object\_class\;\rangle \langle x_{i},y_{i},w_{i},h_{i}\rangle$$

where x and y are the object’s center coordinates, and w and h are the object’s width and height, respectively, with relation to the picture and name of the object class. YOLT’s input dimension is 416 × 416 × 3 for training, but keep in mind that the image size should not be too huge or the important information will be lost. The dataset is passed through learning algorithms and the weights are saved during batch training. The batch size denotes the number of training samples in a single forward pass. The learning rate is utilized to optimize and minimize the neural network’s loss function, which translates the values of variables onto real numbers while simultaneously displaying the associated cost with those values. For training, we specified a maximum batch size of 5000. Momentum is a technique for increasing training speed and precision. This network was tested on eight object classes on a tensor of 26 × 11. Network consists of 26 × 26 grids. For training, we utilized batch size = 64 and filter = 40.

## 5. Results

As discussed in the previous sections, object detection is very important and useful for many applications such as defense and agriculture. We studied from the literature review and found researchers used vehicles for detection using YOLO and SIMRDWN and ships for faster RCNN. For this need, we made a custom dataset for airplanes and performed detection using four architectures: faster RCNN, YOLO, SSD and SIMRDWN. We discussed network training of these four architectures in Section 3. For testing, we used unknown images for detection and measured the accuracy and speed.

The goal of a machine learning model is to generalize patterns in training data in order to predict new data that it has not seen before. When a model adjusts the training data too many times, it perceives patterns that do not exist and performs poorly when predicting fresh data. However, the results show that this does not happen in our case as we used flipping data augmentation techniques so that the models can be adequately trained.

We will discuss network testing of these four architectures one by one.

### 5.1. Network Validation of Faster RCNN

**Figure 10:**
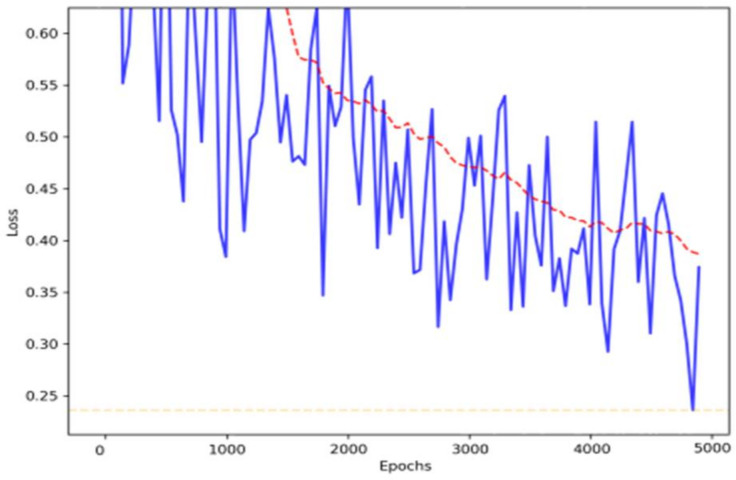
Loss function after training SIMRDWN Model.

We divide the dataset into training and testing parts and check the results on testing part for validation [64,65,66,67]. For detection, Faster RCNN uses a combination of RPN and Fast RCNN with shared convolutional features. Both Fast RCNN and RPN train independently and share convolutional layers in different ways. Therefore, they develop a new technique, which shares the convolutional layers between two RPN and fast RCNN networks rather than training separately (Figure 10). The ROI [68,69,70] layer is responsible for prediction of bounding boxes with acceptance of convolutional features. Faster RCNN takes 1000 × 600 resolution images as input for detection, but its detection time is slow and accuracy is high rather than YOLO and SSD [71,72,73].

Results showed in Table 2 that Faster RCNN was able to identify “aircraft” objects in the dataset with 95.8% accuracy, while only 24 objects were incorrectly categorized. The false discovery rate calculated was 0%, positive prediction was 100%, the true positive rate was 95.8% and the false-negative rate was 4.1%.

### 5.2. Network Validation of YOLO

YOLO uses one network for training and for prediction it also uses one network evaluation for testing of images [74,75,76]. YOLO estimates the class probability for each bounding box and predicts 98 bounding boxes for each image in our dataset. YOLO is fast because it uses a single network for evaluation rather than traditional classifier-based methods. It is a clear grid cell that predicts one box for objects falling in using non-maximal suppression. When the input photos are supplied, YOLO creates a series of bounding boxes and utilizes a non-max suppression approach to identify the proper bounding boxes around the object with maximum intersection over Union [77,78,79,80]. The non-maximal suppression sorts the prediction according to confidence scores and takes one bounding box, which has the highest intersection over the union. YOLO takes 416 × 416 resolution images as input for detection and its detection time is fast. Table 3 shows that YOLO successfully identified “airplane” objects in the dataset with 94.87 percent accuracy, with just 30 objects erroneously classified. The false discovery rate calculated was 0%, positive prediction was 100%, the true positive rate was 94.87% and the false-negative rate was 5.1%.

### 5.3. Network Validation of SSD

**Figure 11:**
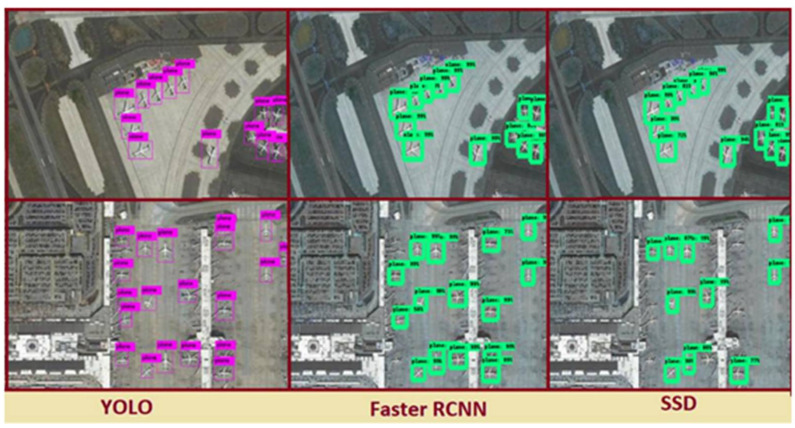
Detection result comparison of faster RCNN, SSD and YOLO.

In SSD, the prediction is simply, by giving an image as input to a single network for evaluation and generates bounding boxes as well as labels. SSD used NMS (Non-maximal suppression) for the removal of duplication [81] (Figure 11). The non-maximal suppression sorts the prediction according to confidence scores and takes one bounding box, which has the highest intersection over union and with the highest probability.

We divide the dataset into training and testing parts and check the results on the testing part for validation. SSD takes 312 × 312 of resolution images as input for detection and its detection time is fast from YOLO and faster RCNN, but its accuracy is low on small objects [82,83,84].

According to the results in Table 4, SSD was able to properly identify “aircraft” items in the dataset with 88.4 percent accuracy, with just 85 objects misclassified. The false discovery rate calculated was 0%, positive prediction was 100%, the true positive rate was 85.4% and the false-negative rate was 14.5%.

### 5.4. Network Validation of SIMRDWN

**Figure 12:**
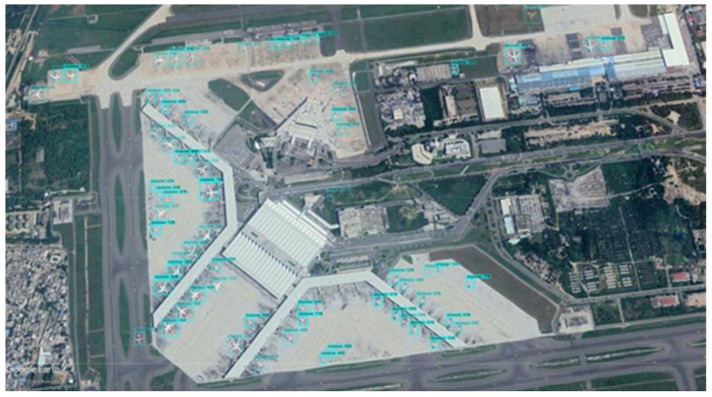
Detection results of SIMRDWN on1920 × 1080 resolution satellite image.

They use two sliding windows with 15% overlap at large image and make cutouts for testing and run classifiers on cutouts and join intelligently returned cutouts according to rows and columns (Figure 12). The backend network for SIMRDWN is YOLO for prediction. Yolo uses one network for training and for prediction, it also uses one network evaluation for testing of images [85,86,87,88,89]. YOLO is fast because it uses a single network for evaluation rather than traditional classifier-based methods. It is a clear grid cell that predicts one box for objects falling in using non-maximal suppression. SIMRDWN takes the highest resolution images for detection [90].

Results showed in Table 5 that SIMRDWN was able to identify “aircraft” objects in the dataset with 97% accuracy, while only 32 objects were incorrectly categorized. The false discovery rate calculated through the SIMRDWN model was 8.8%, the positive prediction was 90%, the true positive rate was 97% and the false-negative rate was 2.94%.

Table 6 shows the accuracy comparisons of different deep learning object detection models used for aircraft detection [91,92,93]. SIMRDWN, faster RCNN, YOLO and SSD have accuracy 97%, 95.31%, 94.20% and 84.61%, respectively. SSD has low accuracy because it uses extra features layers for detection. Due to the usage of extra features layers in SSD some small objects disappear and are not correctly detected [94,95,96,97].

### 5.5. Interface Time

**Figure 13:**
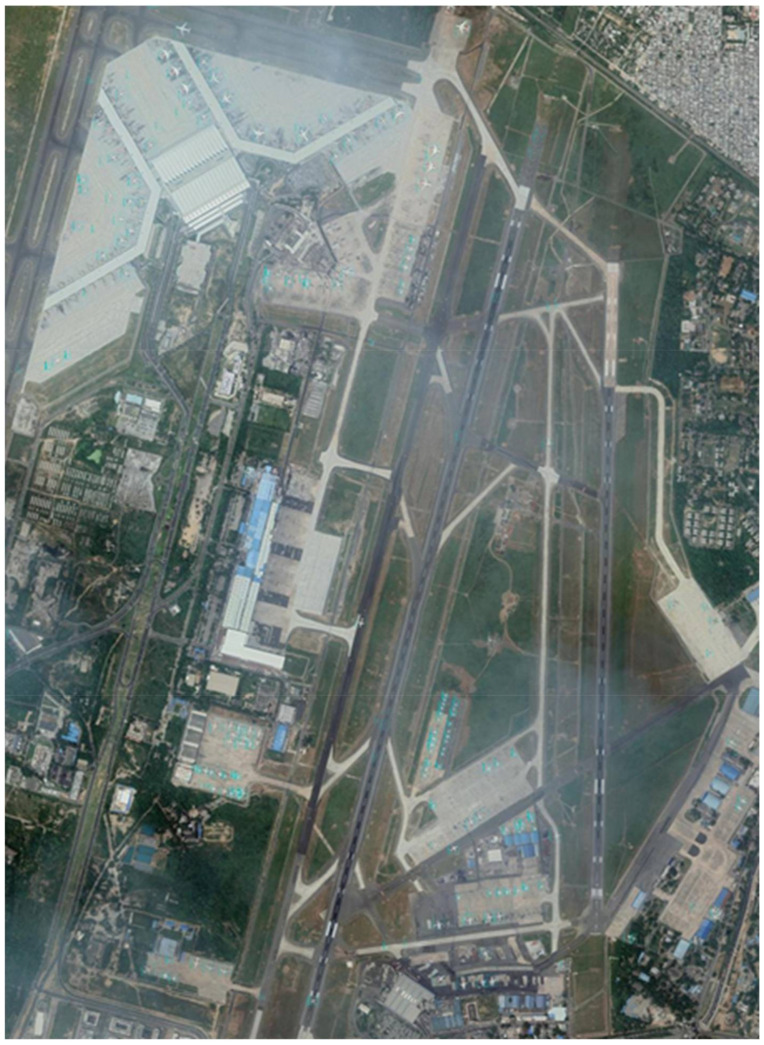
Detection results of SIMRDWN on a 6088 × 8316 resolution satellite image.

Table 7 shows that YOLO has good speed rather than Faster RCNN. The SSD gave the same speed when we tested one image and a batch of ten images for detection [98,99,100,101,102]. It means SSD gives good speed on real-time videos and batches of images (Figure 13). Faster RCNN, YOLO and SSD do not give results when images have the highest resolution (above 1500 1500). SSD has one drawback because it fails on small objects and the drawback of faster RCNN does not perform in real-time. SIMRDWN uses YOLO for detection and it gives good accuracy and speed on highest resolution images [103,104,105,106,107].

## 6. Conclusions

In this paper, four pipelines of object detection are presented, i.e., faster RCNN (faster region-based convolutional neural network), YOLO (you only look once), SSD (single-shot detector) and SIMRDWN (satellite imagery multiscale rapid detection with windowed networks). Detection of small objects in satellite imagery is a very complex problem. As a result, we created a new dataset with extremely small objects in pictures. For training, we utilized 2213 objects and tuned the parameters to compute the anchors for a decent intersection over the union. On our unique dataset, we investigated these four techniques. On unknown satellite pictures with tiny and tightly grouped objects, the models produce good results and fulfill the real-time requirements. The SIMRDWN method for detecting airplanes at crowded airports in satellite images is very reliable, according to the results. SIMRDWN shows 97% accuracy, which is 1.2%, 2.2 and 11.6% more accurate than faster RCNN, YOLO and SSD, respectively.

SIMRDWN is optimized for satellite imagery. However, the analysis also shows us another picture that while it is slightly more accurate than other algorithms, it is much slower than the other algorithms. YOLO is the clear winner when it comes to speed and efficiency. SIMRDWN will fail in the real-time surveillance. When YOLO takes 170 ms to 190 ms, SIMRDWN takes between 5 s to 103 s to execute. This is a drastic difference between the two. In our future work, we will employ YOLO and optimize it to be more efficient and accurate to seamlessly work in real time surveillance.

This research work has some areas which need to be explored in future work. For example, datasets can be increased using synthetic augmentation. More variation can be added in the dataset using generative adversarial networks (GANS). This will help create a more robust validation dataset. As far as model is concerned, Yolo and other models can be further optimized in speed and accuracy.

## Figures and Tables

**Table 1:** Architecture of SIMRDWN (CNN with Maxpooling and convolutional layers).

Type	Filters	Stride	Output
Conv	32	3 × 3	416 × 416 × 32
Maxpooling		2 × 2	208 × 208 × 32
Conv	64	3 × 3	208 × 208 ×64
Maxpooling		2 × 2	104 × 104 × 64
Conv	128	3 × 3	104 × 104 × 128
Conv	64	1 × 1	104 × 104 × 64
Conv	128	3 × 3	104 × 104 × 128
Maxpooling		2 × 2	52 × 52 × 64
Conv	256	3 × 3	52 × 52 × 256
Conv	128	1 × 1	52× 52 × 128
Conv	256	3 × 3	52 × 52 ×256
Maxpooling		2 × 2	26 × 26 × 256
Conv	512	3 × 3	26 × 26 × 512
Conv	256	1 × 1	26 × 26 × 256
Conv	512	3 × 3	26 × 26 × 512
Conv	1024	3 × 3	26 × 26 × 512
Conv	1024	3 × 3	26 × 26 × 1024
Passthrough		10 → 20	26 × 26 × 1024
Conv	1024	3 × 3	26 × 26 × 1024
Conv	Nf	1 × 1	26 × 26

**Table 2:** Confusion matrix of faster RCNN.

Classification	Category	Detection	
		Aircraft	Not Aircraft
Actual	Aircraft Not Aircraft	561 0	24 N/A

**Table 3:** Confusion matrix of YOLO.

Classification	Category	Detection	
		Aircraft	Not Aircraft
Actual	Aircraft Not Aircraft	555 0	30 N/A

**Table 4:** Confusion matrix of SSD.

Classification	Category	Detection	
		Aircraft	Not Aircraft
Actual	Aircraft Not Aircraft	500 0	85 N/A

**Table 5:** Confusion matrix of SIMRDWN.

Classification	Category	Detection	
		Aircraft	Not Aircraft
Actual	Aircraft Not Aircraft	1054 96	32 N/A

**Table 6:** Accuracy comparisons of different deep learning object detection models for satellite imagery.

Sr.	Name	Accuracy	F1-Score
1	Faster RCNN	95.8%	97%
2	YOLO	94.87%	96%
3	SSD	85.4%	91%
4	SIMRDWN	97%	93%

**Table 7:** Detection time of models on different resolution images.

Faster RCNN		YOLO		SSD		SIMRDWN	
Resolution	Time	Resolution	Time	Resolution	Time	Resolution	Time
523 × 315	3.12 s	523 × 315	179.28 ms	523 × 315	3.12 s	4800 × 2718	25.61 s
416 × 416	2.97 s	416 × 416	177.77 ms	416 × 416	2.13 s	1920 × 1080	5.17 s
416 × 416	2.95 s	416 × 416	178.36 ms	416 × 416	2.90 s	8316 × 6088	103.43 s
519 × 323	3.12 s	519 × 323	180.87 ms	519 × 323	2.97 s	4800 × 2718	25.64 s
640 × 360	4.95 s	640 × 360	191 ms	640 × 360	2.96 s	1920 × 1080	5.12 s

## Data Availability

Data are available with the first author and can be shared with anyone upon reasonable request.

## List of Abbreviations

Faster RCNN	Faster region-based convolutional neural network
YOLO	You only look once
SSD	Single-shot detector
AI	Artificial intelligence
CV	Computer vision
NLP	Natural language processing
ML	Machine learning
NN	Neural networks
CNN	Convolutional neural network
YOLT	You only look twice
CONV	Convolutional
VGG	Visual geometry group
ISPRS	International Society for Photogrammetry and Remote Sensing
SIMRDWN	Satellite imagery multiscale rapid detection with windowed networks
